# Successful live birth after transfer of blastocyst and frozen blastocyst from rescue ICSI with application of polarized light microscopy for spindle examination on unfertilized eggs

**DOI:** 10.1186/s13048-015-0150-6

**Published:** 2015-04-08

**Authors:** Jeong Hee Moon, Sara Henderson, Elena Garcia-Cerrudo, Alina Mahfoudh, Shauna Reinblatt, Weon-Young Son

**Affiliations:** Department of Obstetrics and Gynaecology, MUHC Reproductive Center, McGill University Health Center (MUHC), McGill University, Montreal, QC Canada

**Keywords:** Rescue ICSI, Spindle, Polarized light microscopy (PolScope ™), Blastocyst, Vitrification

## Abstract

This article aims to report successful live births after transfer of fresh blastocyst or vitrified/warmed blastocyst derived from intracytoplasmic sperm injection (ICSI) on day-1 of unfertilized mature eggs (so-called “rescue ICSI”) with spindle examination using polarized light microscopy. Two couples who had rescue ICSI performed achieved a positive pregnancy result after the transfer of a fresh or vitrified blastocyst. The two pregnancies led to the live births of a healthy baby boy of 2.72 kg and baby girl of 3.4 kg, respectively.

## Background

Even in patients with normal parameters on semen analysis, total fertilization failure or low fertilization (<20%) in IVF cycles may still occur. This unexpected result may be due to various-factors such as oocyte quality, abnormal sperm, stimulation protocol and disruption of sperm-egg interaction. ICSI on day-1 unfertilized mature eggs (so-called “rescue ICSI”) [1,2] has been recommended in order to result in healthy newborns [3] despite reports of poor quality embryos, higher 3PN rates and a higher incidence of chromosomal abnormalities [4,5]. Low pregnancy rates may also be due to the asynchrony between the developing embryo and the endometrium [6,7]. In a recent report, reducing the time interval between oocyte retrieval and rescue ICSI can decrease oocyte aging [8] and cryopreservation of embryos derived from rescue ICSI can be considered for synchronization to improve the endometrium [6,7]. In addition, it has been reported that spindle examination with polarized light microscopy can help select unfertilized oocytes before rescue ICSI in order to increase the 2PN rate after confirming that no sperm penetrated the egg [9]. In addition, it can decrease the rate of 3PN by avoiding double injection of sperm into unfertilized eggs which have sperm positioned inside the oocyte without leading to activation [9]. This report describes two healthy live births after blastocyst transfer in cases of fertilization failure in IVF cycles in where rescue ICSI was performed after examination of spindle with PolScope™. Although rescue ICSI is still controversial, this treatment is worth considering in order to avoid cycle cancellation.

## Case presentation

### Case 1

**Figure 1:**
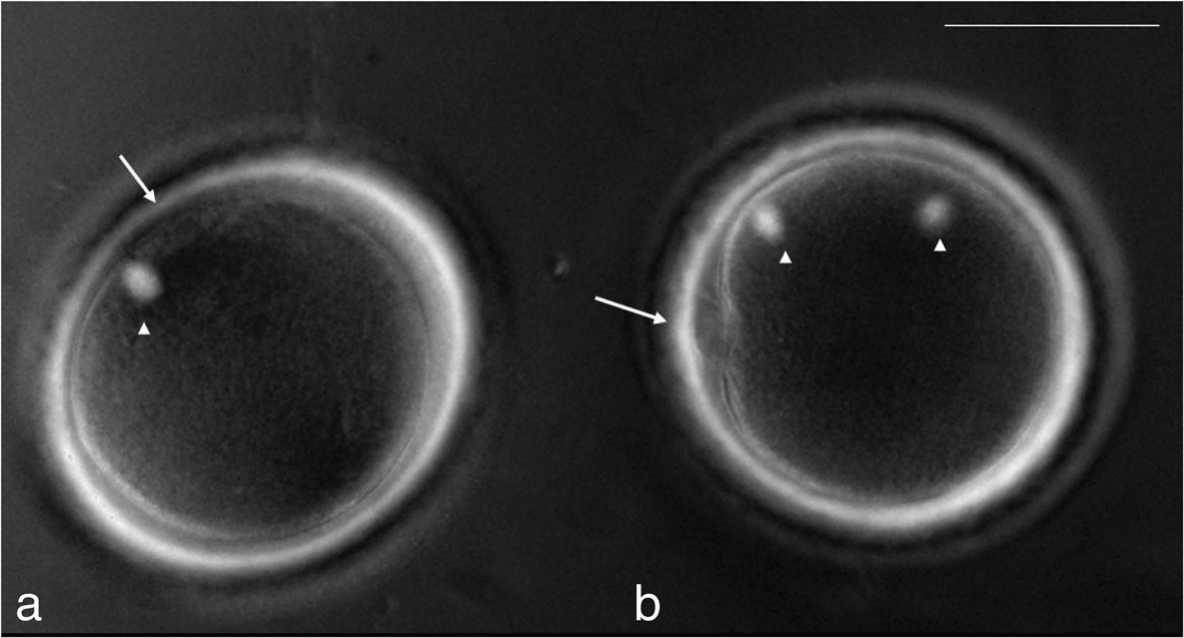
**Unfertilized MII eggs with one-spindle (a) or two-spindle (b) examined with Polscope ™ (arrow; PBs, arrow head; spindles, bar; 50** μ**m).**

The couple, both aged 30, presented with a diagnosis of unexplained infertility after a history of secondary infertility. The couple previously had undergone four artificial uterine inseminations (IUI). Ovarian stimulation was carried out by standard procedures and oocyte retrieval was performed as previously described [10]. Semen was analysed based on standard criterion for IVF for motility, concentration and strict morphology as assessed by World Health Organization (WHO) [11]. Semen analysis showed a normal volume of 2 ml, sperm concentration of 25 million/ml, and 30% progressive motility. The sperm parameters after processing were a volume of 0.5 ml with a concentration of 20 million/ml, 80% motility and 4% normal morphology. Thirteen oocytes were retrieved and were inseminated 3 h later with 100,000 progressive motile spermatozoa/ml. Sperm sample was processed through three-layer density gradients (95%-70%-50%) of Pure sperm (Nidacon, Sweden) followed by two washes in gamete buffer (Cook, Australia). Fertilization was checked 18 h after insemination with an inverted microscope at 20 X under Hoffman modulation contrast. None of the oocytes showed signs of fertilization (i.e., presence of two pronuclei (2PN)) and had insufficient sperm (less than 5 sperm) bound to zona pellucida [8]. Rescue ICSI was promptly performed (21 h after oocyte retrieval) after spindle evaluation to eleven unfertilized mature (MII) eggs that had one spindle with one polar body (PB) visualized (Figure 1a) and one unfertilized MII with two-spindles (Figure 1b) was excluded. Consent from patient was received before injection. Six out of eleven showed 2PN in the afternoon (~6 h after injection) on same day of injection. All zygotes were cultured until day 5 (~116 h) after injection day. One blastocyst was transferred to the patient’s uterus and three blastocysts were cryopreserved for future use using vitrification method. A serum ß-hCG level of 189 IU/L was detected 11 days after embryo transfer. A positive fetal heart beat was confirmed at 6 weeks by a sonography and a healthy boy was delivered vaginally at 38 weeks weighing 2.72 kg.

### Case 2

A 27 year old patient was assessed for IVF-ET due to primary infertility with PCOS. Ovarian stimulation was carried out by standard procedures and oocyte retrieval was performed as previously described [10]. The patient received ovidrel and transvaginal ultrasound-guided oocyte retrieval was performed 35 h later. Eight eggs were retrieved and were inseminated 3 h after oocyte retrieval with sperm concentration of 100,000 per ml. Semen analysis showed a normal volume of 4 ml, sperm concentration of 30 million/ml, and 30% progressive motility. The sperm parameters after processing were a volume of 0.6 ml with a concentration of 40 million/ml, 85% motility and 4% normal morphology. At 17 h post insemination there was no sign of fertilization (2PN) for eight oocytes and all had insufficient sperm bound to the zona pellucida [8]. Eight unfertilized oocytes with the appearance of one spindle and PB were chosen for rescue ICSI (21 h after egg retrieval) which resulted in the fertilization of six eggs in the afternoon on injection day. Embryos were cultured in incubator at 37°C with 6% CO_2_ and 5% O_2_ until blastocyst stage for 5 days (~116 h). One blastocyst was transferred into the female’s uterus and two blastocysts were vitrified for future use. Equilibration medium containing 7.5% (v/v) ethylene glycol (EG) and 7.5% (v/v) 1,2- dimethylsulfoxide (DMSO) and vitrification medium containing 15% (v/v) EG, 15% (v/v) DMSO and 0.5 M sucrose were used for blastocyst vitrification with Cryotop (Kitazato, Japan) vitrification device. Serum ß-hCG level 16 days after egg collection was 27 IU/L and unfortunately resulted in an ectopic pregnancy. The patient returned months later for vitrified-warmed embryo transfer. For warming of vitrified blastocysts, they were first exposed to basic HEPES medium containing 1.0, 0.5, 0.25 M sucrose sequentially and then were washed twice in basic HEPES medium. One blastocyst was transferred into the patient’s uterus following assisted hatching. In this cycle, the patient was treated with oral estrace to prepare the endometrium, which reached adequate thickness (≥8 mm). The phase was complemented by administration of vaginal progesterone. Embryo transfer was performed on day 5 of progesterone administration. Estrogen and progesterone were continued until the pregnancy test. A serum ß-hCG concentration was determined 11 days after embryo transfer. The level of ß-hCG was 1059 IU/L and a positive fetal heart beat was confirmed at 6 weeks by sonography. At 37 weeks, the patient delivered a healthy girl weighing 3.4 kg.

## Discussion

It has been reported previously that unfertilized MII oocytes with only one spindle visualized using PolScope™ after IVF may be due to sperm penetration failure whereas MII oocytes with two spindles results from oocyte activation failure (even if sperm entered the oocytes) [9]. In such cases, performing rescue ICSI as early as possible without any delay to verify appearance of later PN formation may result in embryos with higher viability [8,9]. Recently, in our center, prompt rescue ICSI (16 ~ 18 h after initial insemination) for those eggs with one spindle upon visualization was performed to reduce oocyte aging and avoid the possibility of double insemination. Spindle presence was observed with the polarization microscope and rescue ICSI was performed promptly after checking the spindle in the same glass bottom dish at 37°C [8].When we examined the spindle of unfertilized eggs just before rescue ICSI, there were two other types of spindle appearance examined with PolScope™: zero spindle (no visualization of spindle) and mitotic spindle (our unpublished data). The absence of spindle can be seen during the time of transition from MI to MII associated with low fertilization rate [12,13] or in some unfertilized eggs which lacked signs of spindle presence in the morning but showed 2PN that afternoon without rescue ICSI performed (in our preliminary data not shown). In addition, the mitotic spindle was seen as one big spindle in the middle of the cytoplasm which is thought to be the mitotic plate for embryo development after having missed assessment of 2PN formation but can still produce viable blastocysts (data not shown). Therefore, unfertilized eggs with zero spindle or mitotic spindle were also excluded from rescue ICSI along with those showing two spindles.

Fertilization assessment for oocytes that have undergone rescue ICSI was performed in the afternoon of the same day as injection because it has been suggested that pronuclei for 1-day-old human oocytes inseminated after retrieval can already be seen under the inverted microscope as early as 6 h after ICSI [14]. The embryos could result in good quality blastocysts after culturing for 5 days with the possibility of additional good quality blastocysts for cryopreservation.

It is possible that the disappointing outcomes after rescue ICSI could be due to the asynchrony between embryo development and the endometrial secretory pattern as well as oocyte aging [7]. Therefore, cryopreservation of good quality blastocysts for subsequent transfer in vitrified-warmed cycles could be a good strategy for overcoming poor outcomes due to asynchrony [6].

The main question in cycles with failed fertilization is whether to perform rescue ICSI or to cancel the cycle. After all, the goal of assisted reproductive technology (ART) is to help infertile couples have a healthy baby, not to simply increase the number of transferable embryos. However, previously reported data [15] demonstrates only one out of twenty pregnancies was diagnosed and terminated due to trisomy 21 while the other children were found to be healthy after delivery.

It should also be noted that patients have already invested their time and money for IVF treatment. Therefore, we must also consider the emotional burden that cycle cancellation would have. At our center, the cost of IVF treatment is publicly funded therefore eliminating the financial burden on couples, however single embryo transfers are the standard. On the other hand, there is still the emotional disappointment if cycles are cancelled. We therefore have adopted a policy of culturing to blastocyst stage in order to perform elective single embryo transfer. We suggest that in well-selected patients, rescue ICSI may be considered as an alternative method to avoid cycle cancellation by producing viable blastocysts which lead to normal and healthy babies.

## Conclusions

Early rescue ICSI after spindle examination with PolScope™ could decrease the effects of *in vitro* aging in 1-day-old-unfertilized oocytes and result in a successful pregnancy with the formation of good quality blastocysts for transfer or cryopreservation.

## Consents

Written consent with sign were obtained from patients for publication of this case reports.
